# Supplementary material for: System network analysis of Rosmarinus officinalis transcriptome and metabolome—Key genes in biosynthesis of secondary metabolites
Source: PLoS One. 2023 Mar 2;18(3):e0282316. doi: 10.1371/journal.pone.0282316 (PMC9980811; doi:10.1371/journal.pone.0282316)
Supplement: S1 Table — (DOCX) [file pone.0282316.s001.docx]

**S1 Table. List of protein kinase families and sub families in the selected modules of *R. officinalis***

| Module | PK family | Sub family | Count |
| --- | --- | --- | --- |
| Brown4 | Group CAMK | CAMK_CAMKL-CHK1 (CAMK-Like, Checkpoint Kinase 1) | 2 |
|  |  | CAMK_OST1L (open stomata-like kinase) | 1 |
|  | Group CK1 | CK1_CK1-Pl (Cell Kinase 1, Plant-specific) | 1 |
|  | Group CMGC | CMGC_MAPK (Mitogen Activated Protein Kinase) | 3 |
|  |  | CMGC_GSK (Glycogen synthase 3 kinase) | 1 |
|  | Group Plant-specific | Group-Pl-3 (Group Plant-specific 3) | 2 |
|  | Group RLK-Pelle | RLK-Pelle_DLSV (receptor-like kinase/Pelle, DUF26, SD-1, LRR-VIII and VWA, a moss-specific new RLK subfamily) | 1 |
|  |  | RLK-Pelle_LRR-III (receptor-like kinase/Pelle, leucine-rich repeat-III) | 1 |
|  |  | RLK-Pelle_WAK (receptor-like kinase/Pelle, Wall Associated Kinase) | 1 |
|  | Group TKL | TKL-Pl-3 (Plant-specific 3) | 1 |
|  |  | TKL-Pl-5 (Plant-specific 5) | 2 |
|  |  | TKL-Pl-4 (Plant-specific 4) | 2 |
| Green | Group AGC | AGC_RSK-2 (Ribosomal S6 Kinases 2) | 10 |
|  |  | AGC_NDR (nuclear Dbf2-related kinases) | 1 |
|  |  | AGC_PKA-PKG (Protein Kinase A and Protein Kinase G: cyclic AMP-dependent protein kinase (cAPK) and cGMP-dependent protein kinase) | 1 |
|  | Group CAMK | CAMK_CAMKL-CHK1 (CAMK-Like, Checkpoint Kinase 1) | 5 |
|  |  | CAMK_CDPK (calcium-dependent protein kinases) | 8 |
|  |  | CAMK_AMPK (AMP-activated protein kinase) | 2 |
|  |  | CAMK_OST1L (open stomata-like kinase) | 2 |
|  |  | CAMK_CAMK1-DCAMKL (CAMK family 1, Doublecortin and CaMK-Like) | 1 |
|  | Group CMGC | CMGC_CDK-PITSLRE (Cyclin Dependent Kinase, PITSLRE) | 1 |
|  |  | CMGC_MAPK (Mitogen Activated Protein Kinase) | 3 |
|  |  | CMGC_SRPK (SR Protein Kinase; phosphorylates SR splicing factors) | 1 |
|  |  | CMGC_CDK-CCRK (Cyclin Dependent Kinase, Cell Cycle Regulated Kinase) | 1 |
|  | Group Others | PEK_GCN2 (Pancreatic eukaryotic initiation factor-2alpha kinase, general control non-derepressible) | 1 |
|  |  | WNK_NRBP (With No Lysine (K)' kinases and nuclear receptor binding protein (NRBP)) | 4 |
|  | Group Plant-specific | Group-Pl-4 (Group Plant-specific 4) | 1 |
|  |  | Group-Pl-3 (Group Plant-specific 3) | 1 |
|  | Group RLK-Pelle | RLK-Pelle_WAK_LRK10L-1 (receptor-like kinase/Pelle, Wall Associated Kinase, LRK10-like kinase type 1) | 4 |
|  |  | RLK-Pelle_L-LEC (receptor-like kinase/Pelle, L-type lectin) | 4 |
|  |  | RLK-Pelle_LRR-XII-1 (receptor-like kinase/Pelle, leucine-rich repeat-XII-1) | 8 |
|  |  | RLK-Pelle_DLSV (receptor-like kinase/Pelle, DUF26, SD-1, LRR-VIII and VWA, a moss-specific new RLK subfamily) | 20 |
|  |  | RLK-Pelle_LRR-II (receptor-like kinase/Pelle, leucine-rich repeat-II) | 3 |
|  |  | RLK-Pelle_LRR-VI-1 (receptor-like kinase/Pelle, leucine-rich repeat-VI-1) | 1 |
|  |  | RLK-Pelle_Extensin (receptor-like kinase/Pelle, Extensin) | 4 |
|  |  | RLK-Pelle_LRR-XI-1 (receptor-like kinase/Pelle, leucine-rich repeat-XI-1) | 7 |
|  |  | RLK-Pelle_RLCK-VI (receptor-like kinase/Pelle, Receptor Like Cytoplasmic Kinase-VI) | 3 |
|  |  | RLK-Pelle_WAK (receptor-like kinase/Pelle, Wall Associated Kinase) | 6 |
|  |  | RLK-Pelle_RLCK-VIIa-1 (receptor-like kinase/Pelle, Receptor Like Cytoplasmic Kinase-VIIa-1) | 2 |
|  |  | RLK-Pelle_LRK10L-2 (receptor-like kinase/Pelle, LRK10-like kinase type 2) | 4 |
|  |  | RLK-Pelle_RLCK-VIIa-2 (receptor-like kinase/Pelle, Receptor Like Cytoplasmic Kinase-VIIa-2) | 5 |
|  |  | RLK-Pelle_CrRLK1L-1 (receptor-like kinase/Pelle, Catharanthus roseus RLK1-like) | 1 |
|  |  | RLK-Pelle_PERK-2 (receptor-like kinase/Pelle, Plant External Response Like Kinase 2) | 1 |
|  |  | RLK-Pelle_SD-2b (receptor-like kinase/Pelle, S Domain 2b) | 4 |
|  |  | RLK-Pelle_LRR-Xb-2 (receptor-like kinase/Pelle, leucine-rich repeat-Xb-2) | 1 |
|  |  | RLK-Pelle_LysM (receptor-like kinase/Pelle, LysM Domain-containing Kinase) | 1 |
|  |  | RLK-Pelle_LRR-III (receptor-like kinase/Pelle, leucine-rich repeat-III) | 1 |
|  |  | RLK-Pelle_LRR-VIII-1 (receptor-like kinase/Pelle, leucine-rich repeat-VIII-1) | 4 |
|  |  | RLK-Pelle_LRR-Xb-1 (receptor-like kinase/Pelle, leucine-rich repeat-Xb-1) | 4 |
|  |  | RLK-Pelle_RLCK-XV (receptor-like kinase/Pelle, Receptor Like Cytoplasmic Kinase-XV) | 1 |
|  |  | RLK-Pelle_RLCK-Os (receptor-like kinase/Pelle, Receptor Like Cytoplasmic Kinase-Os) | 1 |
|  |  | RLK-Pelle_RLCK-XI (receptor-like kinase/Pelle, Receptor Like Cytoplasmic Kinase-XI) | 1 |
|  |  | RLK-Pelle_RLCK-IXb (receptor-like kinase/Pelle, Receptor Like Cytoplasmic Kinase-IXb) | 1 |
|  |  | RLK-Pelle_LRR-IX (receptor-like kinase/Pelle, leucine-rich repeat-IX) | 2 |
|  |  | RLK-Pelle_CR4L (receptor-like kinase/Pelle, CRINKLY4-like) | 1 |
|  |  | RLK-Pelle_RLCK-V (receptor-like kinase/Pelle, Receptor Like Cytoplasmic Kinase-V) | 2 |
|  |  | RLK-Pelle_RLCK-X (receptor-like kinase/Pelle, Receptor Like Cytoplasmic Kinase-X) | 1 |
|  |  | RLK-Pelle_LRR-XIV (receptor-like kinase/Pelle, leucine-rich repeat-XIV) | 1 |
|  |  | RLK-Pelle_LRR-VI-2 (receptor-like kinase/Pelle, leucine-rich repeat-VI-2) | 1 |
|  |  | RLK-Pelle_URK-1 (receptor-like kinase/Pelle, Unknown Receptor Kinase 1) | 1 |
|  | Group STE | STE_STE7 (MAP2K (MAP kinase kinase) genes, homologous to yeast Ste 7) | 1 |
|  |  | STE_STE11 (MAP3K (MAP kinase kinase kinase) genes, homologous to yeast Ste 11) | 1 |
|  | Group TKL | TKL-Pl-5 (Plant-specific 5) | 2 |
|  |  | TKL-Pl-1 (Plant-specific 1) | 2 |
|  |  | TKL_CTR1-DRK-2 (CTR1-DRK-2) | 2 |
|  |  | TKL-Pl-4 (Plant-specific 4) | 4 |
|  |  | TKL-Pl-6 (Plant-specific 6) | 2 |
|  |  | TKL-Pl-7 (Plant-specific 7) | 1 |
| Yellow | Group AGC | AGC_RSK-2 (Ribosomal S6 Kinases 2) | 3 |
|  | Group Others | NEK (Mitotic Kinase family, also known as NRK (NimA-Related Kinase, based on Aspergillus NimA)) | 1 |
|  | Group RLK-Pelle | RLK-Pelle_LRR-XIIIa (receptor-like kinase/Pelle, leucine-rich repeat-XIIIa) | 2 |
|  |  | RLK-Pelle_LRR-II (receptor-like kinase/Pelle, leucine-rich repeat-II) | 2 |
|  |  | RLK-Pelle_CrRLK1L-1 (receptor-like kinase/Pelle, Catharanthus roseus RLK1-like) | 2 |
|  |  | RLK-Pelle_RLCK-VI (receptor-like kinase/Pelle, Receptor Like Cytoplasmic Kinase-VI) | 4 |
|  |  | RLK-Pelle_LRR-XIIIb (receptor-like kinase/Pelle, leucine-rich repeat-XIIIb) | 2 |
|  |  | RLK-Pelle_LRR-III (receptor-like kinase/Pelle, leucine-rich repeat-III) | 6 |
|  |  | RLK-Pelle_LRR-VI-1 (receptor-like kinase/Pelle, leucine-rich repeat-VI-1) | 1 |
|  |  | RLK-Pelle_LRR-Xb-1 (receptor-like kinase/Pelle, leucine-rich repeat-Xb-1) | 1 |
|  |  | RLK-Pelle_DLSV (receptor-like kinase/Pelle, DUF26,SD-1, LRR-VIII and VWA, a moss-specific new RLK subfamily) | 4 |
|  |  | RLK-Pelle_LRR-I-2 (receptor-like kinase/Pelle, leucine-rich repeat-I-2) | 1 |
|  |  | RLK-Pelle_LRR-IX (receptor-like kinase/Pelle, leucine-rich repeat-IX) | 2 |
|  |  | RLK-Pelle_PERK-2 (receptor-like kinase/Pelle, Plant External Response Like Kinase 2) | 1 |
|  |  | RLK-Pelle_LRR-VII-1 (receptor-like kinase/Pelle, leucine-rich repeat-VII-1) | 1 |
|  |  | RLK-Pelle_LysM (receptor-like kinase/Pelle, LysM Domain-containing Kinase) | 1 |
|  |  | RLK-Pelle_LRR-XI-1 (receptor-like kinase/Pelle, leucine-rich repeat-XI-1) | 3 |
|  |  | RLK-Pelle_LRR-IV (receptor-like kinase/Pelle, leucine-rich repeat-IV) | 1 |
|  |  | RLK-Pelle_LRR-XI-2 (receptor-like kinase/Pelle, leucine-rich repeat-XI-2) | 1 |
|  |  | RLK-Pelle_CR4L (receptor-like kinase/Pelle, CRINKLY4-like) | 1 |
|  | Group STE | STE_STE20-Fray (MAP4K (MAP kinase kinase kinase kinase) genes, homologous to yeast Ste 20, Fray (Named based on Drosophila family member)) | 1 |
|  | Group TKL | TKL-Pl-4 (Plant-specific 4) | 2 |
