# Supplementary material for: System network analysis of Rosmarinus officinalis transcriptome and metabolome—Key genes in biosynthesis of secondary metabolites
Source: PLoS One. 2023 Mar 2;18(3):e0282316. doi: 10.1371/journal.pone.0282316 (PMC9980811; doi:10.1371/journal.pone.0282316)
Supplement: Attachment — Submitted filename: comment.docx [file pone.0282316.s003.docx]

The manuscript (research article) explores the relationship between crucial secondary metabolites and their biosynthetic genes in the rosemary. The article’s topic fits within the scope of PLOS ONE, and the argument is of interest to a broad audience. However, I have got some comments:

1. In the manuscript, the order of references is chaotic. Please check reference outputs.

2. It seems that the author is unclear about the form of the gene and protein (the name of the gene should be in italics).

3. The author used RT-PCR to confirm the function of hub genes, but only

chose three genes. It is necessary to add more genes to improve the reliability of conclusions.

4. Figures 3, 4, and 5, please, combine them.

5. Line 107: The author pointed out that ‘Many plant species treated with methyl jasmonate are able to produce compounds such as alkaloids, terpenoids, coumarins, and phenolic compounds [2]. On the other hand, the accumulation of rosmarinic acid in cell culture of methyl jasmonate-treated Menta balsamea has been proven [27].’. To our knowledge, the articles about the effect of methyl Jasmonate in Rosemary have been published. Please add relevant literature.

Yao D, Chen Y, Xu X, et al. Exploring the Effect of Methyl Jasmonate on the Expression of microRNAs Involved in Biosynthesis of Active Compounds of Rosemary Cell Suspension Cultures through RNA-Sequencing[J]. International journal of molecular sciences, 2022, 23(7): 3704.

Yao D, Zhang Z, Chen Y, et al. Transcriptome Analysis Reveals Differentially Expressed Genes That Regulate Biosynthesis of the Active Compounds with Methyl Jasmonate in Rosemary Suspension Cells[J]. Genes, 2021, 13(1): 67.
