# Supplementary material for: System network analysis of Rosmarinus officinalis transcriptome and metabolome—Key genes in biosynthesis of secondary metabolites
Source: PLoS One. 2023 Mar 2;18(3):e0282316. doi: 10.1371/journal.pone.0282316 (PMC9980811; doi:10.1371/journal.pone.0282316)
Supplement: Attachment — Submitted filename: comments.docx [file pone.0282316.s004.docx]

The manuscript by Eisa Foroozan et al. aim to reveal the key genes involved in the biosynthesis of secondary metabolites in *Rosmarinus officinalis*. The topic is valuable and is well within the scope of journal. But its main disadvantage is related to the unsuitable method of plant materials collection. Sometimes the text is confusing and some concepts are not clear or correct (see specific comments below). Thus, it must be substantially improved.

My concerns are the following:

1) keywords: It should be listed in alphabetical order.

2) Introduction: I don’t think the opening paragraph (line 43-47) placed here is necessary, which is not closely related to the main point of this paper. Instead, authors should directly introduce the special value of *Rosmarinus officinali*s in the beginning.

3) Line 49: Rosmarinic acid, carnosic acid, carnosol, and essential oils has which important medicinal value should be described more detailedly.

4) Line 54-56: Authors should simplify the statements to improve the readability and scientificity of the paper. My suggestion: Although the secondary metabolites have diverse functions in plant, including regulating growth (citing literature), interacting with the environment (citing literature), and ameliorating abiotic and biotic stress (citing literature), the structure and metabolic pathways of secondary metabolites are still unknown.

5) Plant selection and treatment: The selection of plant materials in this paper is not convincible only based on the previous literatures. Because the results drawn by other researchers cannot be same as yours due to different climate, growth conditions, and treatment methods. Therefore, I suggest authors need supplement the relevant data of the content of rosmarinic acid in *Menta balsamea* under methyl jasmonate treatments.

6) The figures and tables cited in the end of each sentence should be enclosed in brackets (see Line 137, 140, 141, 143 and other places).

7) Line 143: change ‘the major modules’ to ‘the three major modules’.

8) Line 148: Our results revealed that secondary metabolites have significant correlation with the co-expression modules.

9) Line 152-154: ‘While rosmarinic acid… brown4 module.’ I can’t understand what the authors mean. This sentence should be rewritten.

10) Line 157: (Table 2 & 3)

11) Line 166: replace ‘effect’ to ‘affect’.

12) Line 173: Which important family of proteins was identified in all modules except for Yellow?

13) Line 182: It should be Table S1 rather than S1 Table.

14) Line 187: This sentence should be revised as ‘The main systems are listed as follows: Channels/Pores, Electrochemical Potential-driven Transporters, Primary Active Transporters, Group Translocators, Transmembrane Electron Carriers, Accessory Factors Involved in Transport, and Incompletely Characterized Transport Systems.’

15) Authors are responsible for preparing their papers in correct English language. Some sentences are difficult to understand (e.g., Line 193-196), and the manuscript requires substantial grammatical revisions in the present form.

17) Fig. 7: The image resolution is bad. Please export the picture with the suitable format, such as jpg., tiff.

16) Discussion: In general, the discussion is too long and it should be shorten. If authors detect the content of rosmarinic acid in *Menta balsamea*, the discussion can be connected to the results better. Line 209-234, this part is mainly the replication of the results. Line 211: change ‘a disease’ to ‘disease’. The GO enrichment analysis and KEGG enrichment analysis need to add the relevant figures as supplementary materials.

17) Table 4: Don’t make the table on the different pages.

18) Line 547: Miss the title ‘Figure caption’.
